# The Impact of Weekend Catch-up Sleep on Hepatic Steatosis among Adults in the United States

**DOI:** 10.7150/ijms.107214

**Published:** 2025-02-18

**Authors:** Si-Jie Hong, Li-Wei Hong, Xiao-Qin He, Xiao-Hong Zhong, Xue-Qin Zhang

**Affiliations:** 1 Department of Ultrasound, Department of Obstetrics and Gynecology, Women and Children's Hospital, School of Medicine, Xiamen University, Xiamen 361000, Fujian Province, China.; 2 Ministry of Science and Education, Department of Obstetrics and Gynecology, Women and Children's Hospital, School of Medicine, Xiamen University, Xiamen 361000, Fujian Province, China.; 3 Department of Obstetrics, Department of Obstetrics and Gynecology, Women and Children's Hospital, School of Medicine, Xiamen University, Xiamen, China.

**Keywords:** Weekend catch-up sleep, Hepatic steatosis, Obesity, Liver ultrasound transient elastography

## Abstract

**Background:** Short sleep duration significantly increases the risk of liver disease. This study aims to investigate the relationship between weekend catch-up sleep (WCS) duration and hepatic steatosis in adults with varying weekday sleep durations in the United States.

**Methods:** A cross-sectional analysis was conducted using adult participants from NHANES 2017 to March 2020. Anthropometry measurements, clinical features, biochemical parameters, and sleep duration (weekdays and weekends) were recorded. The controlled attenuation parameter (CAP) was utilized to evaluate the degree of liver steatosis. Student's t-test and Mann-Whitney U test were used for unpaired samples. Adjusted multi-variable logistic regression analysis was employed to evaluate the relationship between weekend catch-up sleep and hepatic steatosis.

**Results:** Weekend sleep habits varied based on age, obesity, race, education level, and marital status. Individuals with insufficient weekday sleep (<6 hours) and inadequate WCS (<1 hour) exhibited a significant increase in liver CAP values and a markedly higher probability of hepatic steatosis. In contrast WCS >1 hour effectively reduced the probability of hepatic steatosis. Individuals who adequately compensated for sleep on weekends had lower ratios of AST/ALT, total bilirubin and creatinine levels. Among different BMI populations, WCS was significantly associated with liver health in those with insufficient weekday sleep (<6 hours), whereas in individuals with adequate or sufficient weekday sleep (≥6 hours), WCS only reduced the progression of steatosis in individuals with normal BMI (<25).

**Conclusion:** Adequate weekend catch-up sleep was associated with a lower the incidence of hepatic steatosis in individuals with insufficient weekday sleep duration.

## Introduction

Sleep is a fundamental physiological process crucial for maintaining overall health and well-being. Sufficient duration and quality of sleep are essential for various bodily functions. Despite recommendations from the Sleep Research Society and the American Academy of Sleep Medicine advocating for at least 7 hours of sleep per night, a considerable portion of the American population suffers from sleep deprivation^1^; over 35% of individuals sleep less than 7 hours, with 30% sleeping less than 6 hours^2^, ^3^. In recent years, extensive research has delved into the intricate relationship between sleep patterns and their impact on human health. Studies indicate a close association between short sleep duration and increased susceptibility to hypertension, obesity, cognitive decline, infertility, depression, and mortality^4^-^6^. Another significant area of interest is the connection between sleep and the development of non-alcoholic fatty liver disease (NAFLD).

In response to habitual sleep restrictions, a prevalent sleep pattern known as weekend catch-up sleep (WCS) has emerged, characterized by shorter sleep durations on weekdays compensated by longer sleep durations on weekends. Recent studies suggest that WCS can reduce the heightened inflammatory state associated with short sleep duration during weekdays, effectively alleviating risks related to sleep deprivation, such as obesity, hypertension, insulin resistance, and depression^7^, ^8^. This pattern appears to significantly enhance both physiological and psychological health.

Fatty liver disease (FLD) is a prevalent and severe chronic liver condition worldwide, potentially leading to steatohepatitis, fibrosis, cirrhosis, and even mortality^9^, ^10^. Hepatic steatosis, characterized by excessive intrahepatic lipid accumulation, forms the basis of FLD. Given the lack of effective treatments for FLD, preventing the development of hepatic steatosis is crucial. Controlled attenuation parameters (CAP), measured through Liver Ultrasound Transient Elastography (LUTE), are commonly employed in numerous clinical applications to assess the degree of hepatic steatosis, providing a cost-effective and safe non-invasive tool^11^.

Despite these insights, the association between weekend sleep extension and human liver health remains largely unexplored in the adult population. Therefore, our primary aim is to investigate the relationship between WCS and hepatic steatosis in a nationally representative sample of American adults. By elucidating this relationship, we aim to provide valuable insights into the intricate interplay between sleep patterns and liver health, ultimately informing strategies aimed at mitigating liver diseases.

## Methods

### Study design and population

The National Health and Nutrition Examination Survey (NHANES) is a continuous cross-sectional series of surveys conducted by the US CDC's NCHS, designed to collect data on the health and nutritional status of the non-institutionalized U.S. civilian population, including individuals living in non-institutional group quarters. NHANES uses a complex, stratified, multistage probability sampling process to ensure that the sample is representative of the entire U.S. population. In each survey cycle, primary sampling units are selected from counties across the United States, followed by the selection of secondary units such as households within these areas. Eligible individuals within selected households are then invited to participate. This sampling approach guarantees the inclusion of diverse demographic characteristics, including age, sex, and race/ethnicity. The survey gathers comprehensive data through questionnaires, physical examinations, and laboratory tests to provide a detailed picture of the health status of people across the U.S.

NHANES was approved by the Research Ethics Review Board at the NCHS. Written informed consent was obtained from all participants. As a public-use dataset, this study was exempt from additional review by an institutional review board.

**Figure 1:**
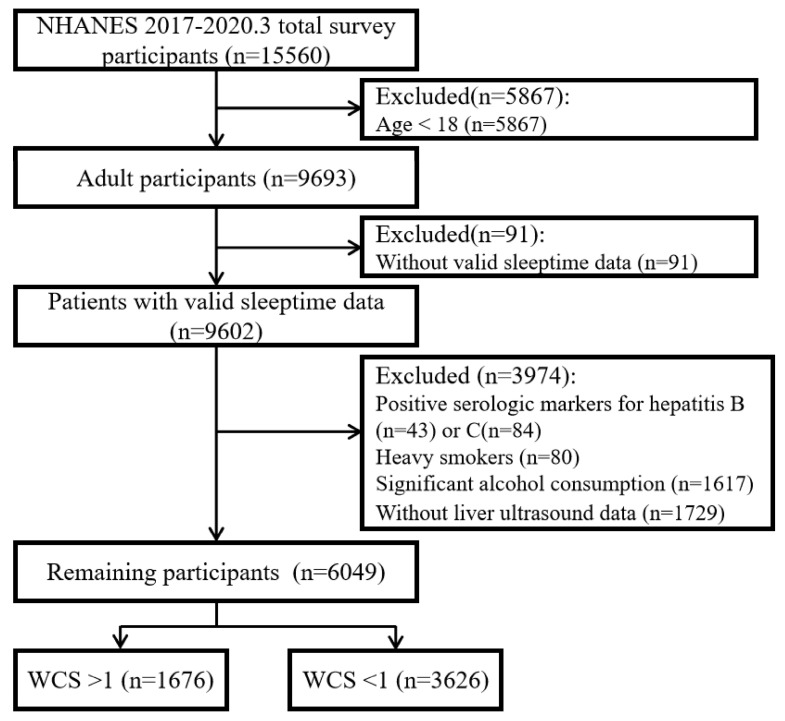
Sample selection process flow chart.

This study analyzed cross-sectional data from a representative sample of United States participants (n=15,560) participating in the 2017-March 2020 pre-pandemic datasets NHANES. We excluded participants who did not undergo VCTE or with incomplete VCTE data (n = 755, due to partial exam, ineligibility or not done), or missing Median CAP scores (n = 1). The exclusion criteria for this study were as follows: (1) Participants under the age of 18; (2) Patients who did not report sleep data; (3) Individuals with positive serological markers for hepatitis B or C virus infection in laboratory data; (4) Heavy smokers (defined as more than 1 cigarette/day); (5) Heavy tobacco users (defined as more than 4 standard drinks/day for men and more than 2 standard drinks/day for women); (6) Participants who did not have valid liver ultrasound transient elastography data. Based on these criteria, the final dataset included 6,049 eligible participants for analysis (Fig. 1).

### Assessments of sleep duration and WCS

In the NHANES survey conducted from 2017 to 2020, participants' sleep durations on weekdays and weekends were determined based on their responses to the following separate questions: (1) Usual sleep time on weekdays or workdays, (2) Usual wake time on weekdays or workdays, (3) Usual sleep time on weekends, and (4) Usual wake time on weekends. Sleep durations on weekdays and weekends were calculated from these responses. The duration of weekend catch-up sleep (WCS) was computed as the weekend sleep time minus the weekday sleep time. Sociodemographic, biochemical, and liver ultrasound data were analyzed between two groups: WCS < 1 and WCS > 1.

### Assessments of steatosis

NHANES 2017-2020 used Liver Ultrasound Transient Elastography (LUTE) to estimate the degree of hepatic steatosis by measuring CAP. VCTE was performed by FibroScan model 502 V2 Touch (Echosens, Paris, France) equipped with a medium (M) or large (XL) probe. Only patients who satisfied the following conditions were considered to complete the assessment of hepatic steatosis: (1) fasted at least 3 hours prior to the exam; (2) able to obtain 10 or more valid measures; (3) the liver stiffness interquartile (IQR)/median ≤30%. CAP values range from 100-400 dB/m, with higher values indicating higher amounts of liver fat. Patients were categorized into three steatosis degrees based on previous studies^12^-^14^: CAP >248, >268 and >280 dB/m as hepatic steatosis stage 1, 2 and 3 respectively.

### Statistical analyses

Statistical analysis was conducted using IBM SPSS 24.0 software (IBM Corp., Armonk, NY, United States). Considering the complexity of the survey method, descriptive statistics were utilized to calculate weighted data. The files "WTINTPRP - Full sample interview weight" and "WTMECPRP - Full sample MEC exam weight" from the 2017-2020 period were used as designated sample weights for this study. To ensure the actual differences between the two groups in comparison, Student's t-test for unpaired samples was used when data followed a normal distribution, while the Mann-Whitney U test was used to compare the groups when the data did not show a normal distribution. Data were presented as mean ± standard error. An adjusted logistic regression model was established to evaluate the relationship between weekend sleep duration (independent variable) and steatosis (dependent variable) under different weekday sleep times. Comparisons between groups were adjusted for Body Mass Index (BMI) using analysis of covariance (ANCOVA). A p-value <0.05 was considered statistically significant.

## Results

### Basic characteristics

To investigate the weekend catch-up sleep (WCS) habits among different populations, we analyzed the basic characteristics of individuals with WCS exceeding 1 hour and those with less than 1 hour. Notable variations were observed between these two groups (Table 1). Individuals with WCS > 1h tended to be younger on average age of 42.02 ± 0.37 years, compared to those with WCS <1h, who had an average age of 51.01 ± 0.31 years. Additionally, the WCS > 1h group had a higher proportion of Mexican American (14.00%), Other Hispanic (12.19%), and Non-Hispanic Black (13.27%) individuals compared to the WCS <1h group (7.24%, 6.76%, and 10.94% respectively), while the proportions of Non-Hispanic White and Non-Hispanic Asian individuals were lower (51.29%, 5.52% vs 63.62%, 7.37%). Moreover, the percentage of individuals with a college education or above was lower in the WCS >1h group (56.79% vs 60.21%). Regarding marital status, fewer individuals were widowed, divorced, or separated (14.19% vs 19.30%), while the proportion of those who had never been married was higher in the WCS >1h group (20.46% vs 17.00%). These differences were statistically significant. However, no significant differences were observed in terms of gender composition and Family Income to Poverty Ratio between the two groups.

### Steatosis development associated with weekend catch-up sleep

**Figure 2:**
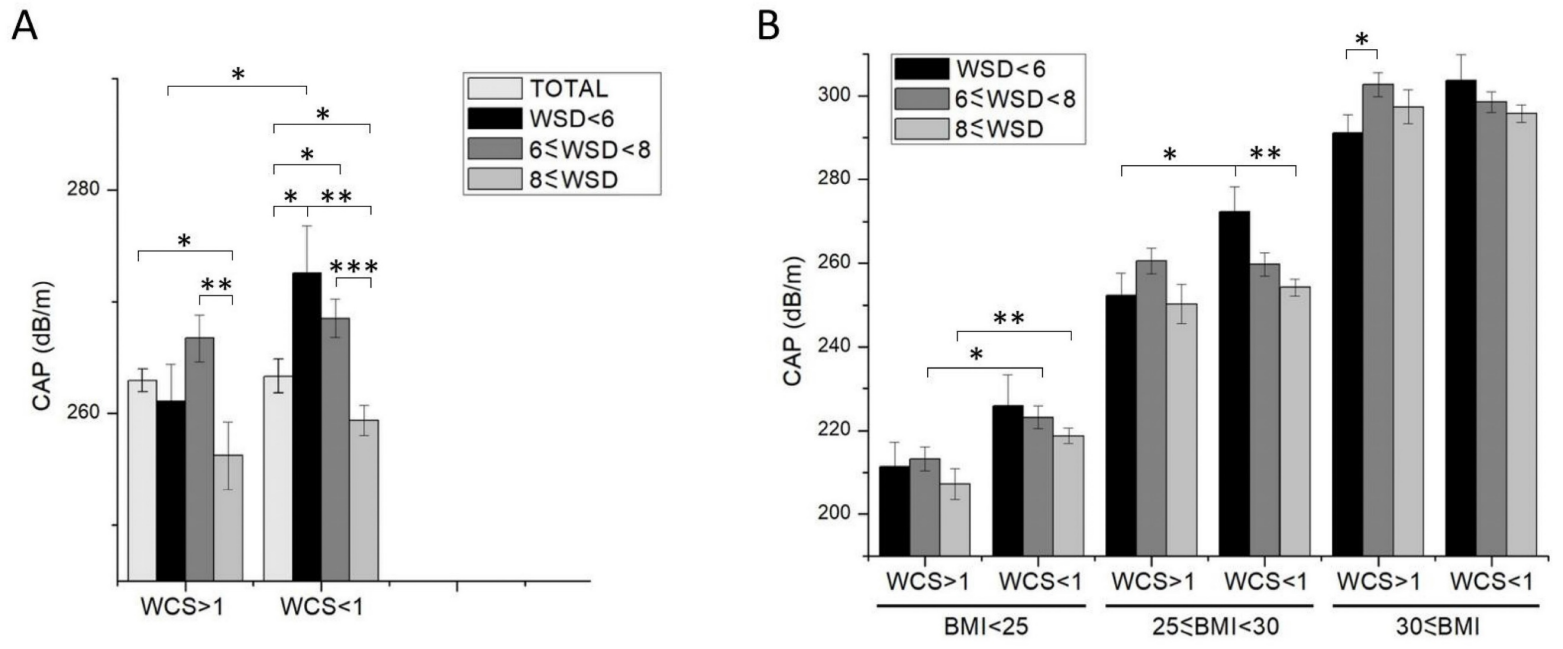
Statistics of the Controlled Attenuation Parameter for Participants in Different WCS Groups. (A) Total Population, (B) Population Grouped by BMI Categories. Data shown are means ± SEM. Asterisks represent statistical significance (*p < 0.05, **p < 0.01 and ***p < 0.001).

To investigate the relationship between weekend catch-up sleep duration and liver health, we analyzed liver ultrasonography data from the NHANES database. Participants were categorized based on their weekday sleep duration into groups with sleep duration of <6 hours, ≥6 to <8 hours, and ≥8 hours. Subsequently, we compared the Controlled Attenuation Parameter (CAP) of liver ultrasonography between individuals with weekend catch-up sleep (WCS) >1h and <1h within each sleep duration group (Fig. 2A). Our findings indicated that a weekday sleep duration of more than 8 hours significantly contributes to a reduction in CAP. Conversely, in patients with shorter weekday sleep duration (< 6 hours) and weekend catch-up sleep of less than 1 hour, there was a significant increase in CAP compared to that with more than 8-hour-weekday sleep duration. However, if the catch-up sleep duration was >1 hour, CAP was comparable in two groups. Furthermore, we classified the degree of liver fat deposition was classified as S1 (>248), S2 (>268), and S3 (>280) based on CAP (Table 2). The results revealed a markedly higher proportion of steatosis in individuals with WCS <1h compared to those with WCS >1h, exhibiting statistically significant differences. This suggests that individuals with short weekday sleep duration who do not obtain sufficient catch-up sleep on weekends are significantly more likely to develop steatosis.

### Biochemical parameters changed in different WCS groups

To further substantiate the association between weekend catch-up sleep and the physical condition of the surveyed population, the biochemical test data of individuals with weekend catch-up sleep durations greater and less than 1 hour was collected and analyzed, including Alanine Aminotransferase (ALT), Aspartate Aminotransferase (AST), Gamma Glutamyl Transferase (GGT), Triglycerides (TG), Albumin, Uric acid, Total Bilirubin, Alkaline Phosphatase (ALP), Creatinine, Creatine Phosphokinase (CPK), Sodium, High-Sensitivity C-Reactive Protein (HS-CRP), Fasting Glucose, and Insulin (Table 3). The biochemical results indicated that in the individuals with weekend catch-up sleep less than 1 hour (WCS<1), the level of C-reactive protein (CRP) was elevated, suggesting inflammation or tissue damage. Additionally, CPK levels were reduced while creatinine levels increased, indicating a potential for muscle inflammation or cardiovascular disease, consistent with previous literature reports. Furthermore, in comparison to those who received sufficient compensation through weekend sleep (WCS>1), the WCS<1h group exhibited a increased AST/ALT ratio and total bilirubin levels, common changes observed in patients with liver injury or dysfunction.

### Relationship between weekend catch-up sleep and steatosis in different BMI group

Previous studies have indicated that obese individuals are prone to developing steatosis, while weekend catch-up sleep contributes to reducing BMI^15^. However, contrary to expectations, the overall data from our study revealed that individuals with WCS >1h had a higher BMI (Table 1). To investigate the relationship between WCS, BMI, and steatosis occurrence, we further stratified the participants based on BMI categories (normal: <25, overweight: 25-30, obese: >30). Our findings demonstrate a significant positive correlation between BMI and controlled attenuation parameter (CAP), consistent with previous literature (Fig. 2B). Within the subgroup of individuals with weekday sleep duration <6 hours, all three BMI groups with WCS >1h exhibited significantly lower steatosis compared to those with WCS <1h. However, among participants with weekday sleep duration between 6-8 hours and >8 hours, WCS >1h only effectively reduced steatosis occurrence in the normal BMI (<25) group, with no significant improvement observed in the overweight (25-30) and obese (>30) BMI groups (Table 4 and Fig. 2B).

### Multivariate logistic regression analysis of weekend catch-up sleep and steatosis

Furthermore, multivariate logistic regression analysis was used to examine the relationship of WCS with steatosis. In the complete sample, the results of the adjusted multivariable regression analysis indicated no significant difference in steatosis prevalence between adults with >1h WCS or <1h WCS (adjusted odds ratio = 1.02, 95% CI (0.89, 1.17), Table 5). Subgroups were created based on weekday sleep duration, and a significantly higher OR for steatosis prevalence was observed in individuals with < 6 hours weekday sleep duration and < 1h WCS (aOR = 1.64, 95% CI = (1.11, 2.43), Table 5).

## Discussion

In today's competitive society, many individuals experience short sleep duration during weekdays^16^. This sleep deficit is primarily compensated for by extending sleep duration over the weekend (Weekend Catch-up Sleep, WCS). Previous studies indicated that catching up on sleep is associated with reduced adverse outcomes of sleep deprivation, and the restoration effect is related to the duration of WCS. Among Korean adults, a higher prevalence of metabolic disorders is associated with increased weekend catch-up sleep^17^. Additionally, Gallup Korea survey reports suggest a correlation between WCS and hypertension prevalence^18^. Ness *et al.* demonstrated that two nights of recovery sleep could restore dynamic lipid responses but not reduce insulin sensitivity induced by five nights of sleep restriction^19^. Han *et al.* reported lower levels of high-sensitivity C-reactive protein (hs-CRP) in the WCS group compared to the non-WCS group^20^, while Pejovic *et al.* found an elevation in plasma IL-6 levels during 24-hour sleep restriction, which returned to baseline after recovery sleep^21^.

These studies suggest that sleep deprivation increases the body's inflammatory response, while WCS reduces the heightened inflammatory state in the blood. Kim *et al.* reported that patients with 1-2 hours of WCS had a lower risk of depression compared to those with WCS < 0 hours. Observations from epidemiological studies have linked short sleep duration to an increased risk of non-alcoholic fatty liver disease (NAFLD)^22^, ^23^. However, the association between Weekend Catch-Up Sleep and human liver health has yet to be investigated. This study fills this gap by analyzing the sleep patterns and levels of hepatic steatosis among participants in the NHANES 2017-2020 cohort.

In this study, we first analyzed the differences among individuals with varying weekend catch-up sleep (WCS) habits and found that these habits differ based on age, obesity, race, educational level, and marital status. Analysis of liver elastography data revealed a significant association between WCS and the prevalence of steatosis in adults with weekday sleep durations of less than 6 hours. Specifically, the statistical analysis of the incidence of hepatic steatosis among participants in different weekend catch-up sleep (WCS) groups revealed that, for individuals with weekday sleep durations < 6 hours, the percentage of S3 hepatic steatosis was 44.74% in the WCS <1 group, compared to 37.82% in the WCS>1 group, showing an almost 7% higher incidence in the former. Logistic regression analysis further indicated that individuals with weekday sleep < 6 hours and WCS <1 hour were 1.64 times more likely to develop hepatic steatosis than those with similar weekday sleep but WCS >1 hour. These findings underscore the importance of adequate weekend sleep in reducing the likelihood of hepatic steatosis among those with insufficient weekday sleep.

Although this study presents novel findings from a representative American population, it has several limitations. Firstly, the current findings cannot be generalized to non-American populations as the association between steatosis and WCS appears to be race-dependent. Secondly, our study relies on self-reported weekday and weekend sleep duration, which may introduce potential recall biases. Thirdly, this is a cross-sectional observational study, therefore, the complex causal relationship between WCS and hepatic steatosis cannot be established, and the correlation should be interpreted as association. Moreover, family background factors have been reported to exert confounding effects on sleep behaviors and health outcomes^24^-^26^. As these factors were not investigated in the current study, the potential interference of family-related influences may introduce biases and errors. Future research is needed in other populations and with longitudinal or intervention designs, using objective sleep measurements to confirm our findings.

In conclusion, we found that weekend catch-up sleep is associated with a lower risk of hepatic steatosis among individuals experiencing insufficient weekday sleep, aiding in both the prevention and management of NAFLD.

## Figures and Tables

**Table 1:** Characteristics of participants according to weekend catch-up sleep duration.

Characteristic	WCS >1 (n=1676)	WCS <1 (n=3626)	*P*-value
Age (years)	42.02 ± 0.37	51.01 ± 0.31	<0.001
Sex			
Male (weighted %)	782 (49.03%)	1735 (47.02%)	0.087
Female (weighted %)	894 (50.97%)	1891 (52.98%)	0.087
Family Income to Poverty Ratio	3.08 ± 0.042	3.04 ± 0.030	0.40
BMI (kg/m^2^)	30.43 ± 0.18	29.49 ± 0.12	<0.001
Race/Ethnicity (weighted %)			
Mexican American	297 (14.00%)	358 (7.24%)	<0.001
Other Hispanic	234 (12.19%)	336 (6.76%)	<0.001
Non-Hispanic White	401 (51.29%)	1335 (63.62%)	<0.001
Non-Hispanic Black	475 (13.27%)	884 (10.94%)	0.0086
Non-Hispanic Asian	190 (5.52%)	530 (7.37%)	0.0043
Other Race	79 (3.73%)	183 (4.06%)	0.28
Education Level (weighted %)			
High school or lower	666 (38.35%)	1486 (36.71%)	0.13
College or above	887 (56.79%)	1973 (60.21%)	0.0096
Marital Status (weighted %)			
Married/Living with Partner	947 (60.49%)	2020 (60.64%)	0.46
Widowed/Divorced/Separated	271 (14.19%)	863 (19.30%)	<0.001
Never Married	335 (20.46%)	578 (17.00%)	0.0015

Data are represented as mean ± SEM. p-Value refers to the comparison between WCS >1h and WCS <1h group.

**Table 2:** Statistical analysis of steatosis incidence among different WCS groups stratified by different weekday sleep duration.

Group	Steatosis	WCS >1	WCS <1	p-Value
Total	≥S1 (n, %)	964 (57.52%)	2055 (56.67%)	0.28
	≥S2 (n, %)	775 (46.24%)	1637 (45.15%)	0.23
	≥S3 (n, %)	663 (39.56%)	1393 (38.42%)	0.21
Weekday sleep < 6h	≥S1 (n, %)	203 (58.17%)	148 (64.91%)	0.051
	≥S2 (n, %)	156 (44.70%)	125 (54.82%)	0.0084
	≥S3 (n, %)	132 (37.82%)	102 (44.74%)	0.049
6h ≤ Weekday sleep < 8h	≥S1 (n, %)	540 (59.15%)	752 (60.89%)	0.21
	≥S2 (n, %)	449 (49.18%)	609 (49.31%)	0.48
	≥S3 (n, %)	386 (42.28%)	530 (42.91%)	0.38
8h ≤ Weekday sleep	≥S1 (n, %)	221 (53.38%)	1155 (53.40%)	0.50
	≥S2 (n, %)	170 (41.06%)	903 (41.75%)	0.40
	≥S3 (n, %)	145 (35.02%)	761 (35.18%)	0.48

p-Value refers to the comparison between WCS >1h and WCS <1h group.

**Table 3:** Biochemical parameters of participants according to weekend catch-up sleep duration.

Characteristic	WCS >1	WCS <1	*P*-value
Alanine Aminotransferase (ALT) (U/L)	24.02 ± 0.40	20.63 ± 0.23	<0.001
Aspartate Aminotransferase (AST) (U/L)	21.80 ± 0.31	20.64 ± 0.17	<0.001
AST/ALT	1.05 ± 0.0098	1.14 ± 0.0069	<0.001
Gamma Glutamyl Transferase (GGT) (IU/L)	27.94 ± 0.71	26.18 ± 0.54	0.06
Triglycerides, refrig serum (mg/dL)	139.43 ± 2.61	140.03 ± 1.86	0.86
Albumin, refrigerated serum (g/L)	41.28 ± 0.081	41.05 ± 0.057	0.023
Uric acid (mg/dL)	5.25 ± 0.036	5.35 ± 0.024	0.023
Total Bilirubin (mg/dL)	0.45 ± 0.0069	0.49 ± 0.0056	<0.001
Alkaline Phosphatase (ALP) (IU/L)	76.13 ± 0.57	76.43 ± 0.42	0.68
Creatinine, refrigerated serum (mg/dL)	0.84 ± 0.0093	0.89 ± 0.0056	<0.001
Creatine Phosphokinase (CPK) (IU/L)	186.12 ± 11.73	154.67 ± 5.97	0.0082
Sodium (mmol/L)	140.58 ± 0.063	140.50 ± 0.045	0.33
HS C-Reactive Protein (mg/L)	3.43 ± 0.12	4.08 ± 0.16	0.0089
Fasting Glucose (mmol/L)	6.14 ± 0.074	6.17 ± 0.045	0.69
Insulin (ųU/mL)	14.53 ± 0.42	14.18 ± 0.59	0.71

Data are represented as mean ± SEM. p-Value refers to the comparison between WCS >1h and WCS <1h group.

**Table 4:** Statistical analysis of steatosis incidence among different WCS groups stratified by different BMI categories and weekday sleep duration.

Group		Steatosis	WCS >1	WCS <1	p-Value
Weekday sleep < 6h	BMI < 25	≥S1 (n, %)	15 (19.5%)	20 (35.1%)	0.022
		≥S2 (n, %)	12 (15.6%)	17 (29.8%)	0.026
		≥S3 (n, %)	8 (10.4%)	9 (15.8%)	0.18
	25 ≤ BMI < 30	≥S1 (n, %)	58 (53.7%)	54 (69.2%)	0.014
		≥S2 (n, %)	43 (39.8%)	40 (51.3%)	0.060
		≥S3 (n, %)	33 (30.6%)	33 (42.3%)	0.049
	30 ≤ BMI	≥S1 (n, %)	128 (80.00%)	72 (80.9%)	0.43
		≥S2 (n, %)	100 (62.5%)	66 (74.2%)	0.026
		≥S3 (n, %)	90 (56.3%)	58 (65.2%)	0.082
6h ≤ Weekday sleep < 8h	BMI < 25	≥S1 (n, %)	40 (17.6%)	69 (24.4%)	0.030
		≥S2 (n, %)	21 (9.3%)	42 (14.8%)	0.025
		≥S3 (n, %)	12 (5.3%)	32 (11.3%)	0.0060
	25 ≤ BMI < 30	≥S1 (n, %)	179 (61.9%)	227 (56.8%)	0.085
		≥S2 (n, %)	141 (48.8%)	179 (44.8%)	0.15
		≥S3 (n, %)	110 (38.1%)	152 (38.0%)	0.49
	30 ≤ BMI	≥S1 (n, %)	319 (81.4%)	450 (83.0%)	0.26
		≥S2 (n, %)	285 (72.7%)	383 (70.7%)	0.25
		≥S3 (n, %)	263 (67.1%)	341 (62.9%)	0.093
8h ≤ Weekday sleep	BMI < 25	≥S1 (n, %)	22 (17.9%)	147 (23.0%)	0.093
		≥S2 (n, %)	11 (8.9%)	91 (14.2%)	0.035
		≥S3 (n, %)	6 (4.9%)	65 (10.2%)	0.010
	25 ≤ BMI < 30	≥S1 (n, %)	57 (47.1%)	344 (51.0%)	0.22
		≥S2 (n, %)	43 (35.5%)	255 (37.8%)	0.32
		≥S3 (n, %)	38 (31.4%)	201 (29.8%)	0.36
	30 ≤ BMI	≥S1 (n, %)	140 (84.3%)	652 (79.2%)	0.053
		≥S2 (n, %)	114 (68.7%)	547 (66.5%)	0.29
		≥S3 (n, %)	100 (60.2%)	488 (59.3%)	0.41

p-Value refers to the comparison between WCS >1h and WCS <1h group.

**Table 5:** Associations between weekend catch-up sleep duration and the risk of hepatic steatosis in clinically relevant subgroups.

GROUP		Steatosis (aOR (95% CI))	p-Value
Total	WCS>1	Reference	-
	WCS<1	1.02 (0.89, 1.17)	0.78
Weekday sleep <6 h	WCS>1	Reference	-
	WCS<1	1.64 (1.11, 2.43)	0.013
6 h ≤ Weekday sleep < 8	WCS>1	Reference	-
	WCS<1	1.04 (0.85, 1.27)	0.69
8h ≤ Weekday sleep	WCS>1	Reference	-
	WCS<1	1.03 (0.81, 1.31)	0.80

p-Value refers to the comparison between WCS >1h and WCS <1h group.

## Abbreviations

NAFLD: Non-Alcoholic Fatty Liver Disease
CAP: Controlled Attenuation Parameter
LUTE: Liver Ultrasound Transient Elastography
BMI: Body Mass Index
ALT: Alanine Aminotransferase
AST: Aspartate Aminotransferase
GGT: Gamma Glutamyl Transferase
TG: Triglycerides
ALP: Alkaline Phosphatase
CPK: Creatine Phosphokinase
HS-CRP: High-Sensitivity C-Reactive Protein
